# Physical activity and physical activity induced energy expenditure in humans: measurement, determinants, and effects

**DOI:** 10.3389/fphys.2013.00090

**Published:** 2013-04-26

**Authors:** Klaas R. Westerterp

**Affiliations:** Department of Human Biology, Maastricht University Medical CentreMaastricht, Netherlands

**Keywords:** doubly labeled water, accelerometer, age, predisposition, exercise training, energy intake, chronic disease, body composition

## Abstract

Physical activity is defined as any bodily movement produced by skeletal muscles that results in energy expenditure. The doubly labeled water method for the measurement of total energy expenditure (TEE), in combination with resting energy expenditure, is the reference for physical activity under free-living conditions. To compare the physical activity level (PAL) within and between species, TEE is divided by resting energy expenditure resulting in a figure without dimension. The PAL for sustainable lifestyles ranges between a minimum of 1.1–1.2 and a maximum of 2.0–2.5. The average PAL increases from 1.4 at age 1 year to 1.7–1.8 at reproductive age and declines again to 1.4 at age 90 year. Exercise training increases PAL in young adults when energy balance is maintained by increasing energy intake. Professional endurance athletes can reach PAL values around 4.0. Most of the variation in PAL between subjects can be ascribed to predisposition. A higher weight implicates higher movement costs and less body movement but not necessarily a lower PAL. Changes in physical activity primarily affect body composition and to a lesser extent body weight. Modern man has a similar PAL as a wild mammal of a similar body size.

## INTRODUCTION

Physical activity is defined as any bodily movement produced by skeletal muscles that results in energy expenditure (Caspersen et al., 1985). There are a large number of techniques for the assessment of physical activity ranging from behavioral observation and self-report to motion sensors. The accepted criterion to validate techniques of estimating habitual physical activity, based on the definition of physical activity, is calorimetry. As such, the doubly labeled water method has become the gold standard for the validation of field methods of assessing physical activity. The doubly labeled water method, applied in humans since 1982, is crucial for the measurement of physical activity-induced energy expenditure (AEE) and for the study of determinants and effects.

Physical AEE is determined by body movement and body size. It requires more energy to move a large body than a small body, one of the reasons why obese people generally move less than lean people. Thus, validating field methods of assessing physical activity against energy expenditure requires adjustment for differences in body size. After adjustment for differences in body size, there are clear differences in the level of habitual activity between subjects. Exercise training is the common way to increase the activity level, where professional athletes reach an energy ceiling in endurance exercise.

Determinants and effects of physical activity cannot always be separated. There is a complicated interaction between physical activity and body weight. Body movement requires energy as produced by muscles. Thus, there is an interaction between physical activity, body weight, body composition, and energy expenditure. To move, one uses muscles and energy as stored in body fat. Excess weight in heavier subjects usually implicates excess body fat, limiting weight-bearing activities like running. In addition to body weight and body composition, physical activity is a function of predisposition, age, and environment. There typically are those that are always on the move and those you cannot get on the move. Additionally, physical activity is a function of physical capacity as affected by energy supply and disease.

The current chapter comprises methods for the measurement of physical activity, followed by sections on determinants and effects of physical activity, with a special focus on the doubly labeled water method.

## MEASUREMENT OF PHYSICAL ACTIVITY

### THE DOUBLY LABELED WATER METHOD FOR THE ASSESSMENT OF TOTAL ENERGY EXPENDITURE

**FIGURE 1:**
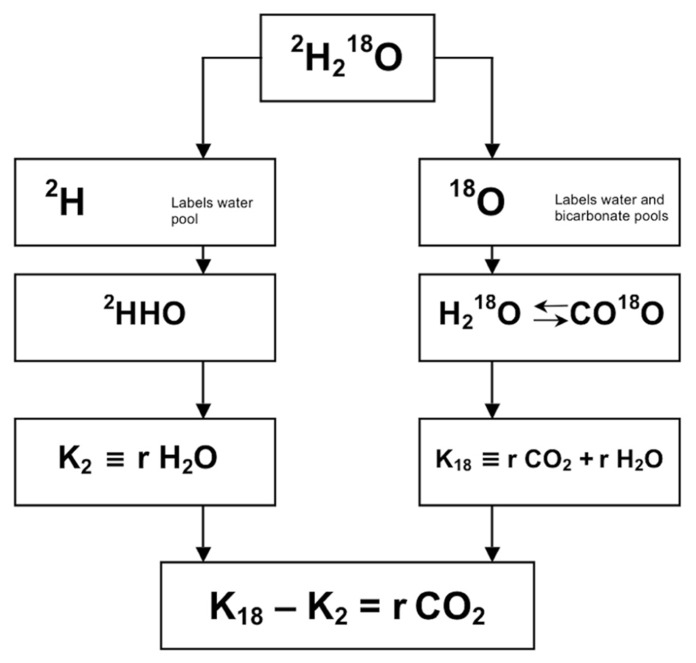
**Principle of measurement of carbon dioxide production with doubly labeled water (^2^H_2_^18^O)**. After administration of water labeled with heavy oxygen (^18^O) and heavy hydrogen (^2^H), the two isotopes mix with the body water, where ^18^O exchanges with CO_2_ in the bicarbonate pools as well. Thus, the elimination rate of ^2^H (K_2_) is a measure for water loss (rH_2_O) and the elimination rate of ^18^O (K_18_) is a measure for rH_2_O plus carbon dioxide production (rCO_2_), and rCO_2_ = K_18_–K_2_.

The doubly labeled water method is a method of indirect calorimetry that was introduced for human use about 30 years ago (Schoeller and Van Santen, 1982). The principle of the method is that after a loading dose of water labeled with the stable isotopes of ^2^H and ^18^O, ^2^H is eliminated as water, while ^18^O is eliminated as both water and carbon dioxide. The difference between the two elimination rates is therefore a measure of carbon dioxide production (**Figure 1**). The deuterium (^2^H) equilibrates throughout the body’s water pool, and the ^18^O equilibrates in both the water and the bicarbonate pool. The bicarbonate pool consists largely of dissolved carbon dioxide, which is an end product of metabolism and passes in the blood stream to the lungs for excretion. The rate constants for the disappearance of the two isotopes from the body are measured by mass spectrometric analysis of samples of a body fluid, blood, saliva, or urine.

The method is developed after the discovery in 1949 that the oxygen atoms in the body water and bicarbonate pools are in equilibration. The method was initially used for studying energy metabolism of small animals in the wild. You capture an animal, administer the dose of labeled water, release the animal and then recapture it after an appropriate interval to assess the rate at which the isotopes disappear from the body. One of the first such studies involved measuring the energy cost of a 500-kilometer flight by trained racing pigeons. It was not until 1982 before the method was first used in people. The reason is that ^18^O-water is expensive and a human requires a much higher dose than a bird. The isotope is not substantially cheaper now, but isotope ratio mass spectrometers have become so sensitive that the method can now work with much smaller doses of isotope. Presently, the method is frequently used with people in several centers.

The method is safe to use in humans as the water is labeled with stable isotopes, ^18^O and ^2^H, at low abundances. Both ^18^O and ^2^H are naturally occurring isotopes, which are present in the body prior to the administration of doubly labeled water. As such, tracer studies depend not on measurement of isotopes concentration, but rather on concentrations in excess of natural abundance or background isotope concentrations. The nominal natural abundances of ^18^O and ^2^H are 2000 and 150 ppm, respectively. Typical doses of doubly labeled water only produce excess isotope abundances of 200–300 and 100–150 ppm for ^18^O and ^2^H, respectively.

The doubly labeled water method can be used to measure carbon dioxide production and hence energy production in free-living subjects for periods of some days to several weeks. The optimal observation period is 1–3 biological half-lives of the isotopes. The biological half-life is a function of the level of the energy expenditure. The optimal observation interval ranges between 3 days for highly active subjects or prematures, respectively, and about 4 weeks in elderly (sedentary) subjects.

An observation starts by collecting a baseline sample. Then, a weighed isotope dose is administered, usually a mixture of 10% ^18^O and 5% ^2^H in, for a 70 kg adult, 100–150 cc water. Subsequently the isotopes equilibrate with the body water and the initial sample is collected. The equilibration time is, depending on body size and metabolic rate, for adults 4-8 h. During equilibration the subject usually does not consume any food or drink. After collecting the initial sample the subject resumes its routines according to the instructions of the experimenter and is asked to collect body water samples (blood, saliva, or urine) at regular intervals until the end of the observation period.

Validation studies resulted in an accuracy of 1–3% and a precision of 2–8%, comparing the method with respirometry. The method has now been applied in subjects at a wide age range and at different activity levels, from premature infants to elderly and from hospitalized patients to participants in a cycle race. The method needs high precision isotope ratio mass spectrometry, working at low levels of isotope enrichment for money reasons mentioned above (Speakman, 1997).

There is still discussion on the ideal sampling protocol, i.e., multi-point versus two-point method. We prefer a combination of both, taking two independent samples at the start, in the midpoint, and at the end of the observation period. Thus an independent comparison can be made within one run, calculating carbon dioxide production from the first samples and the second samples over the first half and the second half of the observation interval (Westerterp et al., 1995).

The doubly labeled water method gives precise and accurate information on carbon dioxide production. Converting carbon dioxide production to energy expenditure needs information on the energy equivalent of CO_2_, which can be calculated with additional information on the substrate mixture being oxidized. One option is the calculation of the energy equivalent from the macronutrient composition of the diet. In energy balance, substrate intake and substrate utilization are assumed to be identical. In conclusion, doubly labeled water is an excellent method to measure energy expenditure in unrestrained humans in their normal surroundings over a time period of 1–4 weeks.

### TOTAL ENERGY EXPENDITURE, ACTIVITY INDUCED ENERGY EXPENDITURE, AND PHYSICAL ACTIVITY LEVEL

**FIGURE 2:**
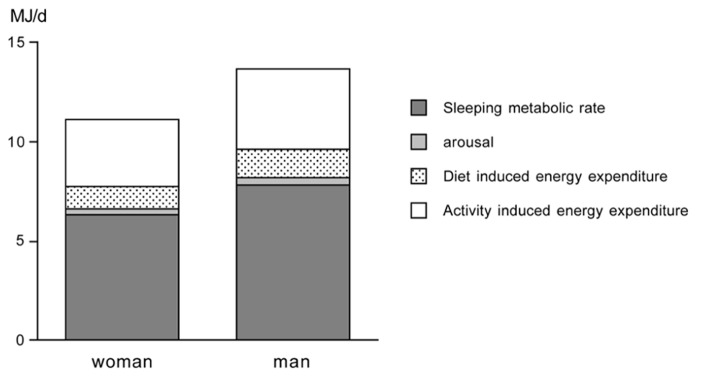
**Components of total energy expenditure for an average young adult woman and man as adapted from Westerterp et al. (1996)**.

Total energy expenditure (TEE) consists of four components, i.e., the sleeping metabolic rate (SMR), the energy cost of arousal, the thermic effect of food or diet-induced energy expenditure (DEE), and the energy cost of physical activity or AEE. Sometimes daily energy expenditure is divided into three components, taking SMR and the energy cost of arousal together as energy expenditure for maintenance or basal metabolic rate (BMR). BMR is usually the main component of TEE (**Figure 2**).

Activity-induced energy expenditure is derived from TEE minus DEE and BMR: AEE = TEE – DEE – BMR. TEE is measured with doubly labeled water as described in the foregoing section. DEE is assumed to be 10% of TEE in subjects consuming the average mixed diet and being in energy balance (Westerterp, 2004). Thus, AEE can be calculated as: AEE = 0.9 TEE – BMR. BMR is measured or estimated with a prediction equation. A measurement of BMR must meet standard conditions of rest, thermoneutrality, fasting, and immobility. The subject must be awake and the measurement must be performed in a thermoneutral environment to avoid heat production or heat loss for maintenance of body temperature. Furthermore the subject must be in the fasted state (absence of DEE) and in rest (absence of AEE). To meet the conditions in practice, measurement of BMR is performed in the early morning. Subjects are instructed to fast overnight before the BMR measurement, and to transport themselves to the research center in a vehicle or bus. They are also asked to avoid exercise the day before testing. Using a ventilated hood system, BMR is measured for 30 min in the supine position. To eliminate effects of subject habituation to the testing procedure, the respiratory measurements during the first 10 min are discarded, and the following 20 min are used to calculate BMR (Adriaens et al., 2003). Alternatively, BMR is estimated with a prediction equation from height, weight, age and gender like the Schofield equations adopted by the FAO/WHO/UNU (2004).

Activity-induced energy expenditure is the most variable component of TEE. To compare AEE between subjects, AEE should be normalized for differences in body size. A frequently used method is expression of AEE per kg body mass, assuming that expenditure associated with physical activity is weight dependent (Schoeller and Jefford, 2002). For comparison of AEE between children and adolescents, AEE is expressed per kg body mass (Hoos et al., 2003) or per kg fat-free mass (Ekelund et al., 2004). Adjusting AEE for fat-free mass is suggested to remove the confounding effect of sex. To compare the physical activity level (PAL) within and between species TEE in MJ/day is divided by BMR in MJ/day, resulting in a figure without dimension: PAL = TEE/BMR. BMR is determined by body size and composition, age and gender. Dividing TEE by BMR adjusts for specific subject characteristics. A larger subject has higher BMR than a smaller subject. TEE is higher as well, and divided by BMR might result in a comparable PAL to a smaller subject.

### LIMITS TO THE PHYSICAL ACTIVITY LEVEL

**FIGURE 3:**
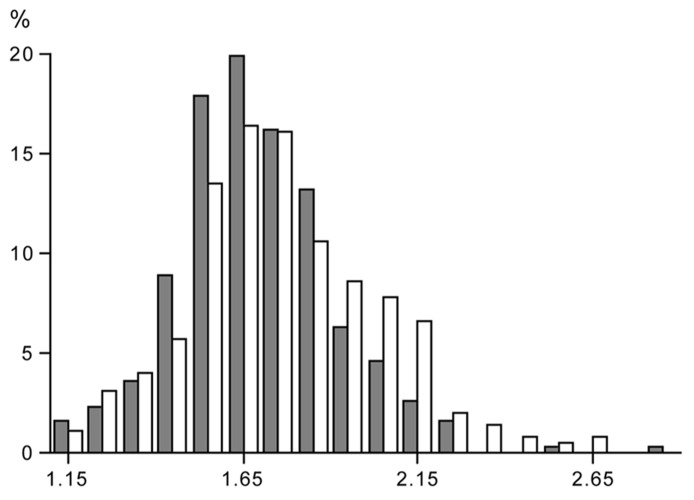
**Frequency distribution of the value of the physical activity level, total energy expenditure as a multiple of basal metabolic rate, in a group of women (closed bars) and men (open bars), where subject characteristics are presented in Table 1**.

Data on free-living energy expenditure, as measured with doubly labeled water, permit the evaluation of limits to the PAL. In our site, data were compiled for more than 600 subjects, where energy expenditure was measured over an interval of 2 weeks with the same protocol (Westerterp et al., 1995). The sample excludes individuals aged under 18 years, or those involved in interventions in energy intake, physical activity including athletic performance, or those that were pregnant, lactating or diseased (**Table 1**). The sample includes similar numbers of women and men, with a wide range for age, height, weight, and body mass index. Despite the wide variation in subject characteristics, there is a narrow range of the PAL of the subjects (**Figure 3**).

**Table 1:** Characteristics of healthy subjects living in Northern Europe, where the physical activity level is measured over 14 days under free-living conditions with doubly labeled water.

Parameter	Women (*n* = 301)		Men (*n* = 346)	
	Mean ± SD	Range	Mean ± SD	Range
Age (y)	42 ± 19	18 – 96	45 ± 19	18 – 96
Height (m)	1.66 ± 0.07	1.49 – 1.86	1.78 ± 0.07	1.60 – 2.04
Body mass (kg)	72 ± 18	40 – 164	84 ± 18	50 – 216
Body mass index (kg/m^2^)	26.2 ± 6.2	14.1 – 55.3	26.4 ± 5.3	15.7 – 61.7
Resting energy expenditure (MJ/d)	6.1 ± 1.0	3.6 – 10.8	7.5 ± 1.1	4.7 – 12.7
Total energy expenditure (MJ/d)	10.3 ± 2.0	4.8 – 18.4	13.2 ± 2.7	6.4 – 22.6
Physical activity level*	1.70 ± 0.23	1.13 – 2.85	1.77 ± 0.28	1.13 – 2.69

* Total energy expenditure as a multiple of resting energy expenditure.

The PAL for “sustained lifestyles” ranges between 1.1 – 1.2 and 2.0 – 2.5 as suggested earlier by Black et al. (1996). There is no sex difference in the PAL. The minimum value of 1.1 – 1.2 is for a subject with no physical activity, TEE being the sum of BMR and DEE. The maximum value of 2.0 – 2.5 is determined by energy intake (Westerterp, 1998). Higher values are difficult to maintain over a long period of time and generally result in weight loss, unless intake is supplemented (see also the section 3.2).

The PAL of a subject can be classified in three categories as defined by the last FAO/WHO/UNU expert consultation on human energy requirements (2004). The physical activity for sedentary and light activity lifestyles ranges between 1.40 and 1.69, for moderately active or active lifestyles between 1.70 and 1.99, and for vigorously active lifestyles between 2.00 and 2.40.

### NON-CALORIMETRIC MEASUREMENT OF PHYSICAL ACTIVITY

There are a large number of non-calorimetric techniques for the assessment of physical activity, which can be grouped into three general categories: behavioral observation, questionnaires (including diaries, recall questionnaires, and interviews), and physiological markers like heart rate and motion sensors. Non-calorimetric techniques of estimating habitual physical activity are needed to study the relationship between physical activity and health. The greatest obstacle to the usage of field methods of assessing physical activity in humans has been the lack of an adequate criterion to which techniques may be compared. The interrelation of various field methods may be of some value, but because there are errors in all methods it is impossible to determine the true validity of any one of them in doing so (Montoye et al., 1996). However, the doubly labeled water method has become the gold standard for the validation of field methods of assessing physical activity (Melanson and Freedson, 1996).

The indicated alternative for doubly labeled water, to assess the PAL of a subject in daily life, is a doubly labeled water validated accelerometer. Accelerometers can be used to study patterns of activity in time. A new generation of accelerometers will provide information on body posture and activity recognition to allow objective assessment of subjects’ habitual activities, options for a healthy change, and effects of the follow-up of any changes (Bonomi and Westerterp, 2012). Simultaneous measurement of body acceleration and heart rate can give information on physical fitness (Plasqui and Westerterp, 2006). Behavioral observation and questionnaires, as a self-report method, can be adequately used as an activity-ranking instrument (Westerterp, 2009).

## DETERMINANTS AND EFFECTS OF PHYSICAL ACTIVITY

### PHYSICAL ACTIVITY LEVEL AND AGE

**FIGURE 4:**
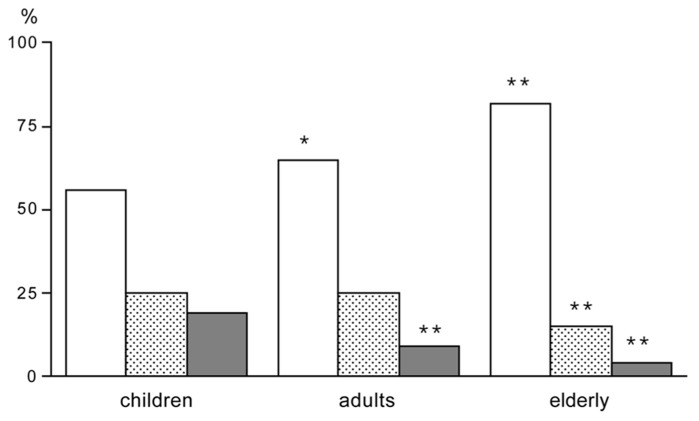
Time spend in activities of low intensity (open bar), moderate intensity (stippled bar) and high intensity (closed bar), expressed as a percentage of the active time, for children, adults and elderly; *P < 0.05 for difference between adults and children; and **P < 0.01 for difference between adults or elderly and children (After Hoos et al., 2004).

Young children have a low PAL. Activity energy expenditure increases from 20% at age one to ~35% at age 18 (Butte et al., 2012). The increase is reflected in the increase of the PAL from 1.4 to 1.75. Activity energy expenditure adjusted for body weight does not show a systematic increase but ranges between about 60 and 80 kJ/kg. It seems young children have a lower activity expenditure and PAL because it takes less energy to move around with a lower body weight. Accelerometers provide information on the activity pattern including activity intensity. Despite the constancy of activity energy expenditure adjusted for body weight from childhood to adulthood, the movement pattern clearly differs. Young children spend more of their active time on high intensity activities (Hoos et al., 2004). Young adults spend on average 9% of their active time on high intensity activities, while the corresponding percentage among the elderly was found to be 4%. In contrast, children spend on average 19% of their total active time on high intensity activities (**Figure 4**). The difference in time spent on high intensity activities between children and adults reflects the different activity patterns among children, which are characterized by short, intermittent bouts of vigorous activity. Probably because of their lower body weight it is easier for children to perform high intensity activities.

Physical activity of an 18-year subject is on average not different from physical activity in a 50-year subject. After age 50, physical activity generally declines, in women as well as men, resulting in a mean PAL of about 1.4 at the age of 90 year (Speakman and Westerterp, 2010). A PAL of 1.4 is the same as the average PAL for a subject staying in a respiration chamber (Westerterp and Kester, 2003). It seems logical that the PAL of a 90-year old is comparable to the PAL for a subject staying all day in a chamber. At age 90, one does not go out very often anymore. The activity pattern of elderly subjects is characterized by low intensity activities (Meijer et al., 2001).

In conclusion, it seems physical activity is the highest at reproductive age.

### PHYSICAL ACTIVITY LEVEL AND EXERCISE TRAINING

**FIGURE 5:**
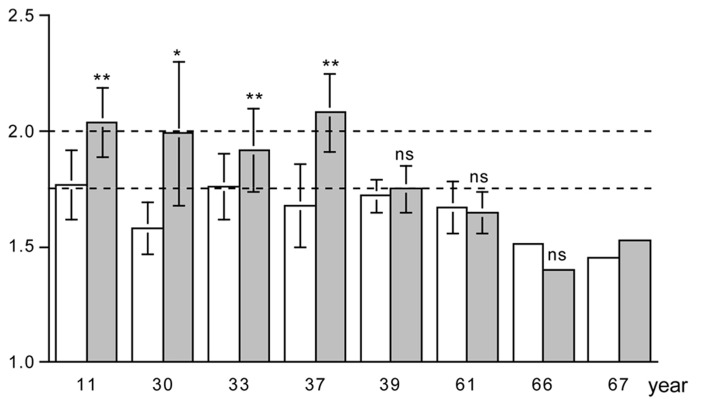
**The physical activity level, total energy expenditure as a multiple of basal energy expenditure, before (open bar) and at the end of a training program (closed bar), for eight studies displayed in a sequence of age of the participants as indicated on the horizontal axis**. The horizontal broken lines denote the average physical activity level of 1.75 and the ceiling value of 2.00 for non-athletes; * *P* < 0.05; and ** *P* < 0.01 for difference with before training program (After Bingham et al., 1989; Blaak et al., 1992; Goran and Poehlman, 1992; Westerterp et al., 1992; Kempen et al., 1995; Van Etten et al., 1997; Hunter et al., 2000; Meijer et al., 2001).

There is a limited number of exercise training studies where the PAL was measured with doubly labeled water, before and at the end of the training intervention. Combining the data of the studies by plotting the PAL in a sequence of the age of the subjects, there are some clear observations to make (**Figure 5**). The PAL before training ranges from lower values around 1.5 in elderly subjects to moderate values around 1.75 in younger subjects. Exercise training induces an increase in physical activity in younger subjects but not in older subjects. The exception is a training study in 39-year subjects; however, in this study training was combined with energy restriction to induce weight loss. In younger subjects, the mean physical activity values reached a ceiling value around 2.0. No training study reported individual PAL values over 2.5. Thus, exercise training induces an increase in physical activity when one is young or middle-aged and eats *ad libitum*.

The lack of an effect of exercise training on the physical activity can only be explained by a compensatory reduction of physical activity in the non-training time. Observations with accelerometers have shown imposed exercise training did not influence spontaneous activity in younger subjects so that their total PALs increased (Meijer et al., 1991; Van Etten et al., 1997). In contrast, elderly subjects compensate for exercise training by a decline in spontaneous physical activity, so that PALs remain unchanged (Meijer et al., 1999).

A potential explanation for a compensatory reduction of physical activity in the non-training time is a negative energy balance. PAL did not increase when exercise training was combined with an energy-restricted diet (Kempen et al., 1995). The PAL in elderly subjects might not respond to exercise training because of a limitation through energy intake, as indicated by a study of the effect of age on energy balance (Ainsli et al., 2002). Exposing 24-year and 56-year subjects to the same strenuous hill walking activity for 10 days resulted in a similar expenditure of about 21.5 MJ/d, where energy intake in the young subjects was with 19.2 MJ/d close to expenditure while the older subjects ate 4 MJ/d less.

The PAL reaches a maximum value of 2.5 times resting energy expenditure in non-athletes. However, professional endurance athletes can reach a value around 4.0 and can maintain this values for several weeks (Westerterp et al., 1986). They are a selection of the population, born to be athletes, training for many years to reach their high level of performance. The training includes exercise and the maintenance of energy balance at a high level of energy turnover. The latter implicates the supplementation of the diet with energy drinks. Highly trained athletes have learned to eat the maximum amount of food during hard physical work (Sjödin et al., 1994).

### PHYSICAL ACTIVITY AND PREDISPOSITION

**FIGURE 6:**
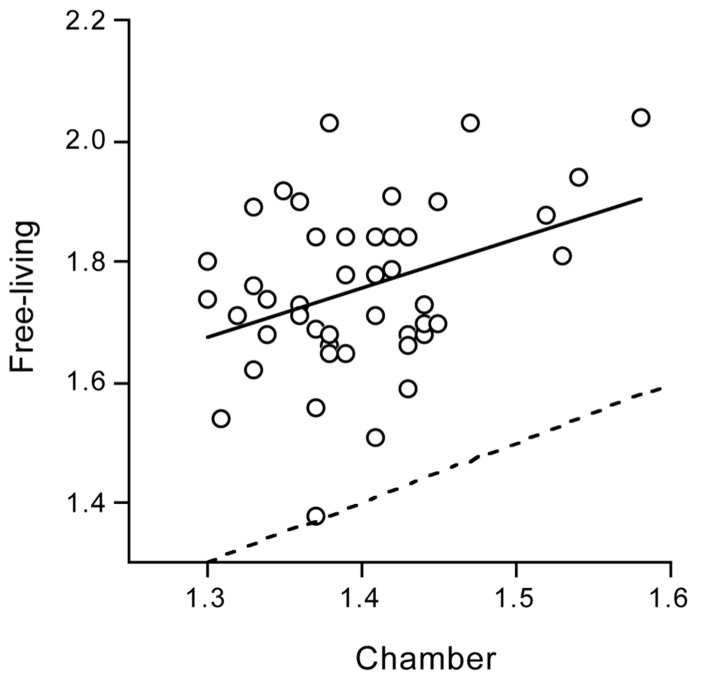
**Free-living physical activity level plotted as a function of physical activity level in the confined environment of a respiration chamber, with the line of identity (dotted) and the linear regression line (continuous) (After Westerterp and Kester, 2003)**.

Some can quietly sit and read for hours while others do not have the perseverance to be quiet. The “between subjects variation” in physical activity is large as reflected in the doubly labeled water assessed PAL under daily life conditions in non-athletes (**Figure 3**). Surprisingly, between subjects variation in physical activity is also large within the identical confined space of a respiration chamber, indicating an effect of predisposition (Westerterp and Kester, 2003). The mean PAL of the subjects in the chamber was 1.40 ± 0.06, on the lower end of the frequency distribution (**Figure 3**) as expected. However, the minimum value was as low as 1.30 and the maximum value as high as 1.58. There was a subject with an AEE of 1.0 MJ/d and a similar sized subject spending 3.0 MJ/d in AEE. Subjects with a relatively low or high PAL in the respiration chamber turned out to be, respectively, relatively sedentary or physically active in free-living conditions as well (**Figure 6**). Further studies, as described below, provided evidence for an important genetic component in the threefold variation in AEE among individuals in the same confined environment of a respiration chamber and the significant relation with PAL in free-living conditions.

The test for a genetic contribution was based on a classic twin design. Intrapair differences in monozygotic twins are due to environmental factors and measurement errors, whereas intrapair differences in dizygotic twins are additionally affected by genetic factors. Physical activity was measured over two consecutive weeks with a doubly labeled water validated tri-axial accelerometer for the measurement of movement. Subjects were 20 same-sex twin pairs, including similar numbers of monozygotic and dizygotic twins, age 25 ± 7 year, and not living together. The PAL was significantly related within twin pairs and the relation was nearly twice as strong within monozygotic than within dizygotic twins. The calculated contribution of genetic factors to the variance in physical activity was 72–78% (Joosen et al., 2005). Thus, a large part of the variation in physical activity between subjects can be ascribed to predisposition. The relatively high contribution of a genetic component to variation in physical activity does not automatically imply subjects with high predisposition for a sedentary life style are less active than subjects with a predisposition for an active life style. The ultimate activity level is the outcome of an interaction between genes and environment. It only takes more effort for subjects with a predisposition for a sedentary life style to reach the same activity level as for those with predisposition for an active life style.

### PHYSICAL ACTIVITY LEVEL AND BODY WEIGHT

Physical activity implies displacement of the weight of a body part like arms, legs, or the full body. Together with activity duration and intensity, body weight determines the variation in AEE. The effect of body weight on physical activity is illustrated by activity changes during growth from birth to adult weight, physical activity and underweight in anorectic subjects, and physical activity and overweight in obese subjects. Body weight increases from three to four kg at birth to an adult value of 60 to 70 kg. Activity energy expenditure adjusted for body weight does not show a systematic increase, as explained in section 3.1. Children can spend more of the active time in high intensity activities than adults (**Figure 4**), as it takes less energy to move the smaller body.

In adults, underweight and overweight is often associated with hyperactivity and hypo-activity, respectively. By monitoring body movement in addition to the measurement of TEE with doubly labeled water, it was shown the paradoxical hyperactivity in anorexia nervosa only occurs in subjects with a higher body mass index (Bouten et al., 1996). The average PAL was not different between a group of women with anorexia nervosa and a control group. However, when subjects were assigned to low, moderate and high levels of daily physical activity, a u-shaped distribution was found for the women with anorexia while control subjects were normally distributed with respect to different activity levels. The u-shaped distribution in women with anorexia was related to the body mass index of the subjects, with relatively low body mass index values corresponding to low levels of physical activity and high body mass index values corresponding to high levels of physical activity. Subjects with a relatively low body mass index had low levels of physical activity and spent less time on activities like sports and exercise, and more time on activities like standing, lying, or sitting than subjects with a higher body mass index. This is in accordance with the reduction in physical activity in the course of chronic energy deficiency and human starvation (see section 3.5). Physical activity decreases as a consequence of malnutrition and declining physical capacity.

**FIGURE 7:**
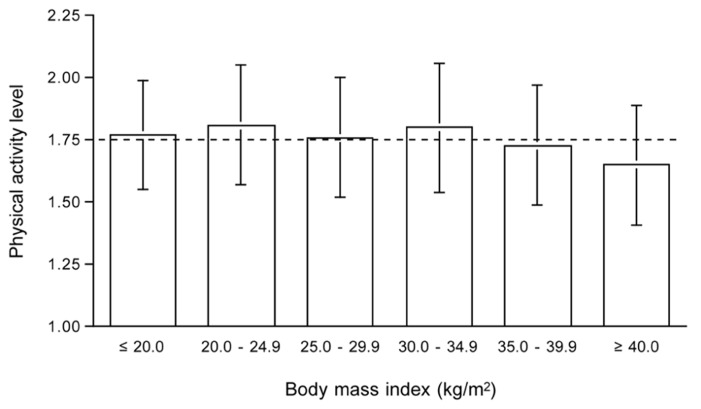
**Physical activity level by body mass index category for subjects aged 18 to 50 year from Table 1**. The horizontal broken line denotes the average physical activity level of 1.75.

Overweight and obesity is not associated with a lower PAL. Activity energy expenditure is similar or even higher in heavier subjects. Only in subjects with a body mass index higher than 35 kg/m^2^, PAL values are reduced (Prentice et al., 1996). Selecting young adults, age range 18–50 year, from our own database as presented in **Table 1**, leads to the same conclusion (**Figure 7**). The average PAL is around 1.75 for all body mass index categories except the very highest. The average value for subjects with a body mass index of 40 kg.m^2^ or higher (*n* = 12) was 1.65 ± 0.24.

A study in adolescents from the same school showed AEE was similar for obese and gender matched control subjects (Ekelund et al., 2002). The fact that AEE is similar and not proportionally higher in subjects with a higher body weight has consequences for body movement. Indeed body movement, as measured simultaneously with accelerometers, was lower in obese than in normal-weight subjects. Overweight implies less physical activity, that is less body movement, but because of the larger body weight, the decreased movement still results in similar energy expenditure as subjects with a normal bodyweight. In conclusion, a higher weight implies less body movement as shown by the typical occurrence of high intensity activity bursts in young children before reaching adult weight. Overweight subjects are less physically active than normal-weight subjects despite physical activity-related energy expenditure is not necessarily lower.

### PHYSICAL ACTIVITY AND ENERGY INTAKE

There are several studies on the effect of overfeeding and underfeeding on physical activity as measured under free-living conditions with doubly labeled water. The effect of overfeeding on physical activity, calculated by expressing TEE as a multiple of resting energy expenditure is non-significant (Westerterp, 2010). There does not seem to be an effect of overfeeding on physical activity, when overfeeding is lower than twice maintenance requirement, as observed in studies lasting up to 9 weeks.

Long-term underfeeding clearly affects physical activity as already shown by the Minnesota experiment (Keys et al., 1950). It was initiated to determine the effects of relief feeding, necessitated by the famine in occupied areas of Europe during World War II. Normal weight men were subjected to 24-weeks of semi-starvation, followed by rehabilitation. The weight maintenance diet of 14.6 MJ/d was reduced to 6.6 MJ/d during semi-starvation. In the 24 weeks of semi-starvation, body weight went down from an average of 69 to 53 kg. At the end of the 24-week interval, subjects reached a new energy balance as body weight leveled off at the lower value. Energy expenditure equalled energy intake, i.e., energy expenditure went down from 14.6 MJ/d to 6.6 MJ/d, a reduction of 55%. The largest saving on energy expenditure could be ascribed to a decrease in activity energy expenditure (**Table 2**). Subjects were not capable of doing anything more than hanging around. More recent underfeeding studies were generally performed in overweight and obese subjects, not reducing body weight as much below normal values as in the Minnesota experiment. Then, underfeeding does not seem to affect PAL though there are indications for a reduction, not persisting in time (Westerterp, 2012).

**Table 2:** Energy saved by 24 weeks semi-starvation in the Minnesota Experiment (Keys et al., 1950).

	MJ/d	% of total	
Basal metabolic rate	2.6	32	65% for decreased active tissue; 35% for lower tissue metabolism
Diet-induced energy expenditure	0.8	10	
Activity-induced energy expenditure	4.7	58	40% for reduced body weight; 60% for reduced physical activity
Total	8.0	100	

There are many comparative studies on the effect underfeeding and the effect of underfeeding in combination with exercise training. The general conclusion is that underfeeding is an effective method to lose weight and that there is little effect of an additional exercise-training program. Explanations for a non-existent effect of the addition of exercise to an energy-restricted diet are a low compliance to the exercise prescription and/or a negative effect of exercise training on dietary compliance. Another explanation for a non-existent effect on weight loss of the addition of exercise to an energy-restricted diet is derived from a typical study performed in Maastricht (Kempen et al., 1995). Obese women were randomly assigned to diet alone or diet and exercise for 8 weeks. The exercise group participated in aerobic and fitness exercises, in three 90-min sessions per week, supervised by a professional trainer. Daily energy expenditure decreased similarly in the diet group and the diet plus exercise group from 12.3 to 10.8 MJ/d and from 12.1 to 11.0 MJ/d, respectively. The PAL was the same for the two groups, before as well as at the end of the intervention. Exercise training did not induce an increase in AEE as observed in subjects with *ad libitum* food intake. Subjects compensated for the training activity with a decrease in physical activity during the non-training time.

### PHYSICAL ACTIVITY LEVEL AND DISEASE

**FIGURE 8:**
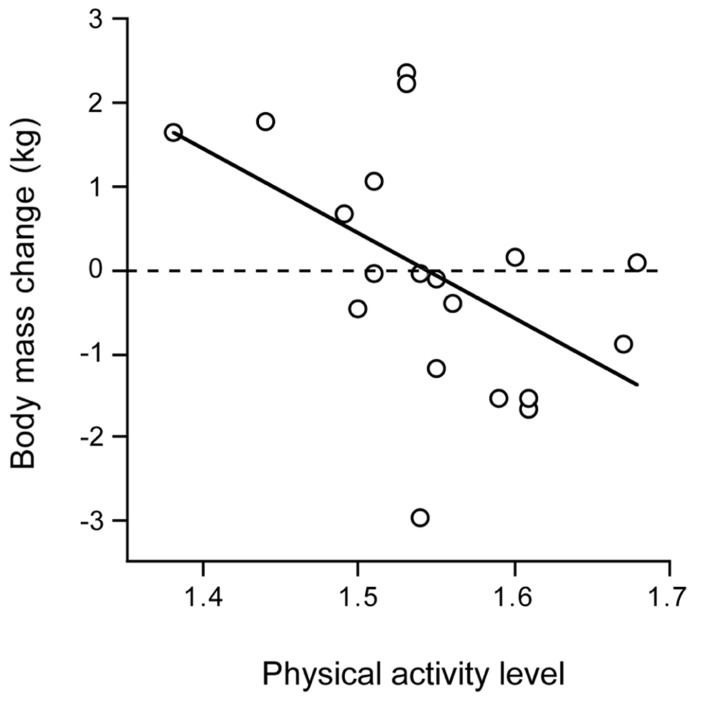
**Body mass change in patients with chronic obstructive pulmonary disease over three months after clinical rehabilitation, plotted as a function of the physical activity level (After Goris et al., 2003)**.

Chronic disease negatively affects physical activity, here illustrated by observations in patients with chronic obstructive pulmonary disease (COPD). COPD is associated with muscle wasting, a decrease in respiratory muscle strength and endurance and impaired physical fitness. Patients with COPD often suffer from weight loss due to an inadequate dietary intake combined with increased energy expenditure. Physical activity, as the main determinant of variation in energy requirement, may play an important role. TEE in COPD is elevated, which can be primarily attributed to the activity component. Interestingly, there is no difference in TEE between COPD patients with normal resting energy expenditure and those with increased resting energy expenditure (Baarends et al., 1997). Patients with normal resting energy expenditure appeared to have higher energy expenditure for activities than those patients with COPD who had increased resting energy expenditure. The PAL was significantly higher in the former group than in the latter group. Physical activity affects the energy need of the COPD patient and determines energy balance. In depleted ambulatory outpatients with COPD, energy balance could be reached with oral nutritional supplements as a function of physical activity. Weight change was negatively associated with the energy requirement for physical activity (**Figure 8**). Patients with a PAL above 1.55 lost weight and with a PAL below 1.55 gained weight (Goris et al., 2003). The disease appears to be an important limitation for an active lifestyle. Chronic disease reduces physical activity and physical capacity, possibly through a limited energy supply.

### PHYSICAL ACTIVITY AND BODY COMPOSITION

The interrelation between physical activity and body composition is based on comparisons between subjects and within subjects. In a between subject design, body composition is compared between subjects with a lower and higher activity level. The question is whether body composition differs between sedentary and physically active individuals. In a within subject design, body composition is compared within subjects before and after an activity intervention. Then, the question is whether body composition changes when one gets less active or more active. Both analyses are described; starting with a comparison between subjects followed by a description of the effect of changes in activity behavior on body composition within the same individual.

The comparison of body composition between subjects with a lower and higher activity level was conducted in a cohort of 529 subjects, included in the compiled data presented in **Table 1** (Speakman and Westerterp, 2010). The analysis showed that at the population level, differences in body composition are generally not related to differences in physical activity. Increasing age is associated with a lower PAL, higher fat mass and lower fat-free mass. For the same body weight, body composition is different at older ages than at younger ages, i.e., fat mass is higher and fat-free mass is lower in the elderly. However, the age-induced reduction of physical activity does not seem to be directly related to the age-induced increase in fat mass and decrease in fat-free mass. At any age, body mass does not systematically differ between a sedentary and a more physically active subject.

**FIGURE 9:**
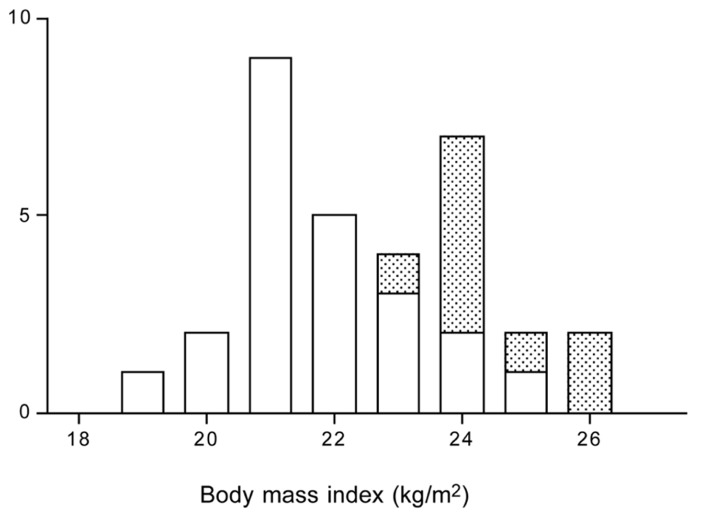
**Frequency distribution of the body mass index of subjects that successfully trained to run a half marathon (open bars) and of the dropouts (stippled bars), the latter were 9 out of 32 subjects (After Westerterp et al., 1992)**.

**FIGURE 10:**
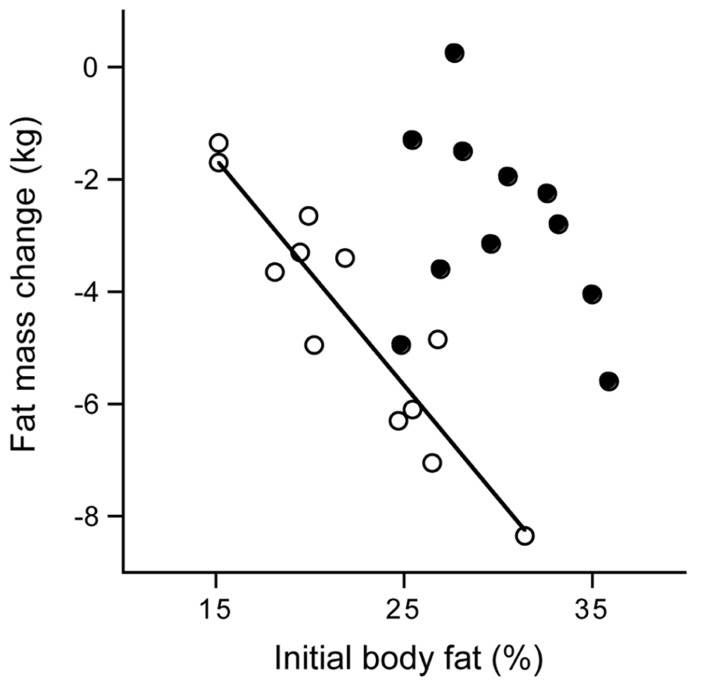
**Fat mass change from before until 40 weeks after the start of a training period to run a half marathon plotted versus the initial body fat percentage for women (closed dots) and men (open dots) with the calculated linear regression line for men (After Westerterp et al., 1992)**.

Many studies show changes in body composition in response to a change in physical activity through exercise training. In young adults, long-term endurance training induces an increase in fat-free mass and, when available, a decrease in fat mass. The latter effect is especially pronounced in men. Here, as an example, a training study in 37-year subjects as included in **Figure 5**. The study included a training program of nearly 1 year in preparation of running a half marathon (Westerterp et al., 1992). Subjects were sedentary men and women who did not participate in any sport like running or jogging and who were not active in any other sport for more than 1h/week. Out of nearly 400 respondents to an advertisement, 16 women and 16 men were selected, between the ages of 30–40 years old, with a normal body weight. The

latter implied a body mass index, based on self reported weight and height, between 20 and 25 kg/m^2^. During the study, five women and four men withdrew because they were unable to keep up with the training program. It appeared all dropouts were in the heaviest category with a body mass index of 23 kg/m^2^ or higher (**Figure 9**). The observation implies that it is difficult to keep up high-intensity training with a higher body weight, especially training involving weight displacement like running. Surprisingly, successful subjects did not lose weight. Apparently, the exercise training-induced increase in energy requirement eventually increased hunger. One has to eat more to maintain the additional training activity, especially in the long-term. The 11 women finishing the 40-week training lost on average 2 kg fat and gained 2 kg fat-free mass. The 12 men that finished the training lost on average 4 kg fat and gained 3 kg fat-free mass. For men, the change in body fat was highly related to the initial fat mass. That is, subjects with a higher initial percentage body fat lost more fat than those who were leaner at the start. This was not so for women (**Figure 10**). Body fat can be reduced by physical activity although women tend to compensate more for the increased energy expenditure with an increased intake, resulting in a smaller effect compared with men. Women tend to preserve their energy balance more closely than men. Women especially do not lose much body fat, even when a high exercise level can be maintained.

The increase in fat mass with increasing age is not prevented through a physically active lifestyle (Westerterp and Plasqui, 2009). Young adults were observed over an average time interval of more than 10 years. Physical activity was measured over two-week periods with doubly labeled water and doubly labeled water validated tri-axial accelerometers, and body fat gain was measured with isotope dilution. There was a significant association between the change in physical activity and the change in body fat, where subjects with higher activity level at the start were those with a higher fat gain at follow up after more than 10 years. A physically active lifestyle inevitably results in a larger decrease of daily energy expenditure at later age than a sedentary lifestyle. A change to a more sedentary routine does not induce an equivalent reduction of energy intake, even in the long-term, and most of the excess energy is stored as fat. Thus, it seems difficult to overcome the loss of fat-free mass and the gain of fat mass with increasing age.

### PHYSICAL ACTIVITY LEVEL OF MODERN MAN

Energy expenditure of modern man is generally thought to be low. People increasingly adopt sedentary lifestyles in which motorized transport, mechanized equipment, and domestic appliances displace physical activities and manual work. Few people are employed in active occupations and leisure time is dominated by sedentary activities behind a computer or watching television. On the other hand, the main part of variation in physical AEE between individuals can be ascribed to genetics, as described in section 3.3. It is unlikely that the genetic background has changed. Changes through natural selection take tens of generations, especially for features like physical activity, determined by many genes. Additionally, in the current society with an abundant food supply, there is no selection pressure in favor of a low physical activity, i.e., low energy expenditure, that otherwise would be necessary to limit energy requirement.

The PAL of modern man is put in perspective, based on analysis of measurements with doubly labeled water (Westerterp and Speakman, 2008). Three tests were performed. Firstly, changes in PAL, as derived from TEE and resting energy expenditure, were compiled over time (**Table 1**). Secondly, PAL in modern Western societies were compared with those from third world countries mirroring the physical activity in Western societies in the past. Thirdly, levels of physical activity of modern humans were compared with those of wild terrestrial mammals, taking into account body size and temperature effects.

**FIGURE 11:**
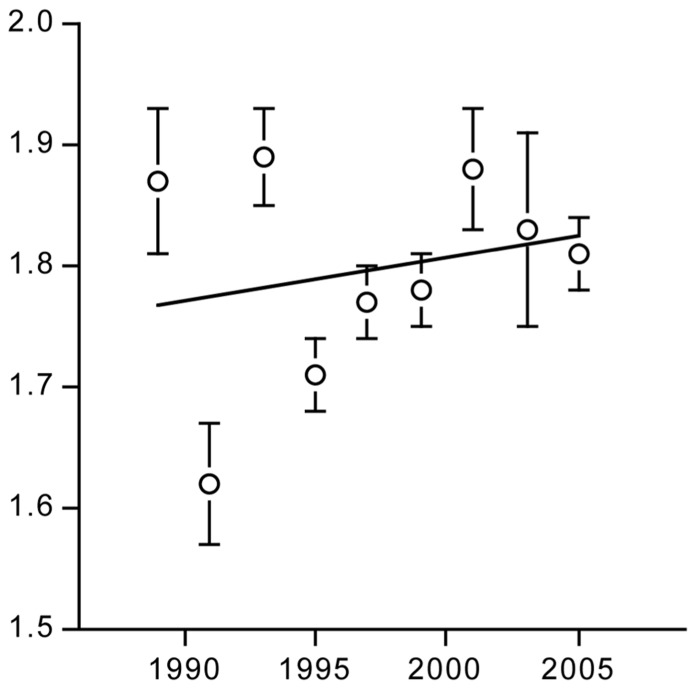
**Time trend of the physical activity level for a population around Maastricht in the Netherlands (After Westerterp and Speakman, 2008)**.

The PAL slightly increased over time (**Figure 11**), indicating physical activity did not decrease during the two decades where rates of obesity doubled in the Netherlands. Compiled literature data from North America, where obesity rates tripled over the same time interval, also suggested the PAL increased rather than decreased. PAL in rural third world countries were not different from individuals of Western societies.

**FIGURE 12:**
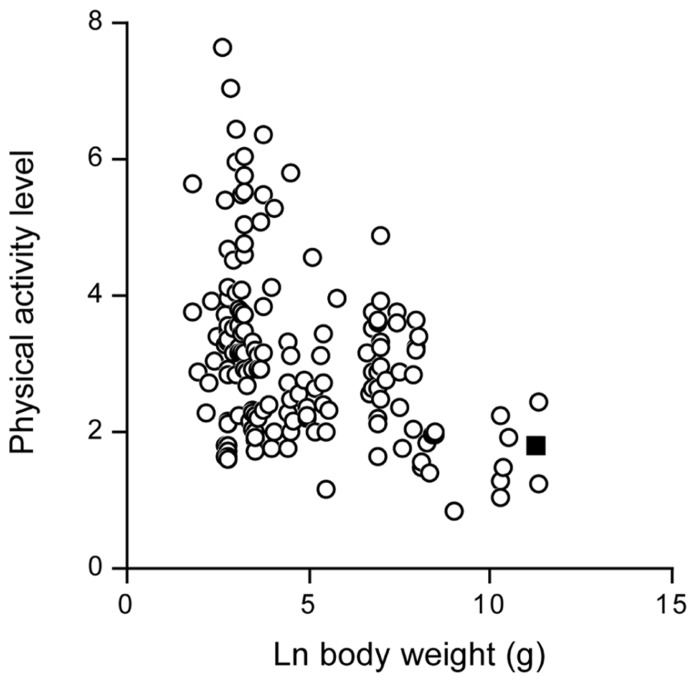
**The physical activity level in wild terrestrial mammals, plotted as a function of body weight**. The value for modern man is indicated as a closed square (After Westerterp and Speakman, 2008).

The doubly labeled water method started with studying energy metabolism of animals in the wild. Since then, data on more than 90 different terrestrial mammal species have been published. Body sizes range from 20-gram mice to wild red deer weighing over 100 kg. For many wild mammals measures of TEE are made at ambient temperatures below the thermoneutral zone. Thus, the PALs for these mammals reflect the combination of activity expenditure and the energy spent on thermoregulation. In fact the PAL calculated as TEE divided by basal energy expenditure is negatively related to body weight (**Figure 12**) reflecting the increasing thermoregulatory load as body size declines. Hence the PAL for contemporary humans is at the lower end of the distribution of activity level values when the effects of body mass are ignored, in line with the previous findings, but they are at exactly the expected level, once the effect of body weight on the PAL is taken into account.

In conclusion, a free-living mammal close to the body size of man has a comparable activity level to humans. The PAL of modern man is in line with a free-living wild mammal.

## DISCUSSION

Physical activity, defined as any bodily movement produced by skeletal muscles that results in energy expenditure, is derived from measurement of energy expenditure. Doubly labeled water is an excellent method to measure energy expenditure in unrestrained humans over a time period of 1-4 weeks. AEE and PAL is derived from TEE and measured or estimated BMR as described in section 2.2. Alternatively, physical activity can be derived from the residual of the regression of TEE on total body water, where total body water is derived from the dilution spaces of deuterium and O^18^. BMR is a function of fat-free mass and total body water is a measure for fat-free mass. Thus, differences in total body water reflect differences in BMR.

Activity-induced energy expenditure is the most variable component of TEE and is determined by body size and body movement. The effect of body size on AEE is corrected for by expressing AEE per kg body mass or by expressing TEE as a multiple of BMR. The expression of TEE as a multiple of BMR is precluded when the relation between TEE and BMR has a non-zero intercept (Carpenter et al., 1995). Then, TEE can be adjusted for the effect of body size in a linear regression analysis.

The indicated non-calorimetric method to assess physical activity is a doubly labeled water validated accelerometer (section 2.4). Validation studies of accelerometers with doubly labeled TEE as a reference should be critically evaluated. The largest component of TEE is BMR, as shown by the frequency distribution of PAL values in **Figure 3**, where most PAL values are below 2.5. A PAL value below 2.5 denotes AEE is less than 50% of TEE (Westerterp, 2003). BMR, as the largest component of TEE, can be estimated from height, weight, age, and gender. Thus, prediction equations of TEE based on height, weight, age, and gender usually show a high explained variation. Adding accelerometer output to the equation as an independent variable, often does not explain any additional variation (Plasqui and Westerterp, 2007). The indicator for the validity of an accelerometer is the increase in explained variation or the partial correlation for accelerometer output, not always presented.

Evidence was presented for age, exercise training, predisposition, body weight, energy intake, and disease as determinants of PAL. A decrease of physical activity with increasing age and an increase of physical activity with exercise training affect body composition and to a lesser extent body weight. The fact that a free-living mammal, close to the body size of man, has a comparable level of energy turnover, i.e., a comparable level of physical AEE to humans, indicates that the energy we spend on physical activity lies in the normal range. It may well be that obese individuals seem to behave rather sedentary, but as soon as their weight-bearing activity takes place, they spend a very large amount of energy on activity because of their well known large bearing of body weight.

## Conflict of Interest Statement

The author declares that the research was conducted in the absence of any commercial or financial relationships that could be construed as a potential conflict of interest.
